# Separable MSE-Based Design of Two-Way Multiple-Relay Cooperative MIMO 5G Networks

**DOI:** 10.3390/s20216284

**Published:** 2020-11-04

**Authors:** Donatella Darsena, Giacinto Gelli, Ivan Iudice, Francesco Verde

**Affiliations:** 1 Department of Engineering, University of Naples Parthenope, I-80143 Naples, Italy; donatella.darsena@uniparthenope.it; 2 National Inter-University Consortium for Telecommunications (CNIT), I-80125 Naples, Italy; gelli@unina.it; 3 Department of Electrical Engineering and Information Technology, University of Naples Federico II, I-80125 Naples, Italy; 4 Italian Aerospace Research Centre (CIRA), I-81043 Capua, Italy; ivan.iudice@unina.it

**Keywords:** amplify-and-forward (non-regenerative) relays, minimum-mean-square-error criterion, multiple-input multiple-output (MIMO) systems, optimization, two-way relaying

## Abstract

While the combination of multi-antenna and relaying techniques has been extensively studied for Long Term Evolution Advanced (LTE-A) and Internet of Things (IoT) applications, it is expected to still play an important role in 5th Generation (5G) networks. However, the expected benefits of these technologies cannot be achieved without a proper system design. In this paper, we consider the problem of jointly optimizing terminal precoders/decoders and relay forwarding matrices on the basis of the sum mean square error (MSE) criterion in multiple-input multiple-output (MIMO) two-way relay systems, where two multi-antenna nodes mutually exchange information via multi-antenna amplify-and-forward relays. This problem is nonconvex and a local optimal solution is typically found by using iterative algorithms based on alternating optimization. We show how the constrained minimization of the sum-MSE can be relaxed to obtain two separated subproblems which, under mild conditions, admit a closed-form solution. Compared to iterative approaches, the proposed design is more suited to be integrated in 5G networks, since it is computationally more convenient and its performance exhibits a better scaling in the number of relays.

## 1. Introduction

Cooperative multiple-input multiple-output (MIMO) communication techniques, wherein data exchange between MIMO terminal nodes is assisted by one or multiple MIMO relays, have been studied for Long Term Evolution Advanced (LTE-A) cellular systems [1,2,3], since they assure significant performance gains in terms of coverage, reliability, and capacity. Relay technology has been also considered for Internet of Things (IoT) applications, by allowing in particular the support of the massive access for fog and social networking services [4,5,6]. One of the main changes when going from LTE-A to 5th generation (5G) systems is the spectrum use at radically higher frequencies in the millimeter-wave (mmWave) range [7]. However, mmWave signals are highly susceptible not only to blockages from large-size structures, for example, buildings, but they are also severely attenuated by the presence of small-size objects, for example, human bodies and foliage [8]. In this regard, cooperative MIMO technology additionally represents a possible approach for circumventing the unreliability of mmWave channels [9] in 5G networks.

**Figure 1:**
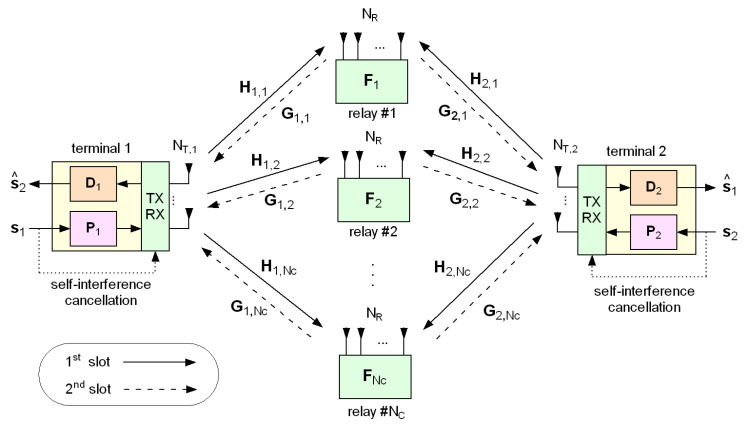
Model of the considered two-way relaying multiple-input multiple-output (MIMO) 5G network.

In addition, 5G systems have stringent requirements in terms of spectral efficiency. Many relaying protocols operate in *half-duplex* mode [10,11,12,13], where two time slots are required to perform a single transmission, due to the inability of the relays to receive and transmit at the same time. To overcome the inherent halving of spectral efficiency, a possible remedy for 5G applications is to adopt *two-way* relaying [14] (see Figure 1), which works as follows: (i) in the first slot, the two terminal nodes simultaneously transmit their precoded signals to the relays; (ii) in the second slot, the relays precode and forward the received signals to the terminals. Since each terminal knows its own transmitted signal, the effects of self-interference can be subtracted from the received signal at the terminals, and the data of interest can be decoded. On the other side of the coin, with respect to the one-way relaying setting, the optimization of two-way cooperative networks is complicated by fact that terminal precoders/decoders and relay forwarding matrices are coupled among themselves.

Design and performance analysis of two-way cooperative MIMO networks encompassing multiple *amplify-and-forward* (AF) or *non-regenerative* relays has been considered in References [15,16,17,18,19]. Compared with the single-relay case [20], the multiple-relay scenario generally leads to more challenging *nonconvex* constrained optimization problems, which are usually solved by burdensome iterative procedures. In Reference [15], by adopting a weighted sum-mean-square-error (MSE) or a sum-rate cost function, iterative gradient descent optimization algorithms are proposed, with transmit-power constraints imposed at both the terminals and the relays. A similar scenario is considered in Reference [16] and Reference [17]. In Reference [16], the original constrained minimum sum-MSE nonconvex optimization problem is iteratively solved. Specifically, the algorithm of Reference [16] starts by randomly choosing the terminal precoders and the relay forwarding matrices satisfying the transmission power constraints at the source terminals and the relay nodes. In each iteration, the terminal precoders, the relay forwarding matrices, and the decoders are alternatingly updated in Reference [16] through solving convex subproblems: first, with given precoders and relay forwarding matrices, the optimal decoders are obtained in closed-form by solving an unconstrained convex problem; second, with fixed precoders and decoders, the relaying matrix of all the relays are updated in closed-form one-by-one by freezing the relaying matrices of the other relays; finally, given the decoders and relaying matrices, the precoders are updated by solving a convex quadratically constrained quadratic programming problem. A different iterative optimization procedure is proposed in Reference [17], based on the matrix conjugate gradient algorithm, which is shown to converge faster than conventional gradient descent methods. Finally, some recent papers [18,19] propose architectures for two-way relaying based on relay/antenna selection strategies.

In this paper, we propose an optimization algorithm for two-way AF MIMO relaying 5G networks, where terminal precoders/decoders and relay forwarding matrices are jointly derived under power constraints on the transmitted/received power at the terminals. Rather than attempting to solve it iteratively, we derive a relaxed version of the original minimum sum-MSE nonconvex optimization, which allows one to decompose it in two separate problems that admit a closed-form, albeit suboptimal, solution. We show by Monte Carlo trials that our closed-form approach performs comparably or better than representative iterative approaches proposed in the literature for the same scenario with a reduced computational complexity, especially for increasing values of the number of relays.

## 2. Network Model and Basic Assumptions

We consider the two-way MIMO 5G network configuration of Figure 1, where bidirectional communication between two terminals, equipped with $N_{T,1}$ and $N_{T,2}$ antennas, respectively, is assisted by $N_{C}$ half-duplex relays, each equipped with $N_{R}$ antennas. We assume that there is no direct link between the two terminals, due to high path loss values or obstructions. Even though our approach can be generalized, for simplicity, the considered physical layer is that of a single-carrier cooperative system where all the channel links are quasi static and experience flat fading.

Let $s_{1}\in C^{N_{S,1}}$ and $s_{2}\in C^{N_{S,2}}$ denote the symbol vectors to be transmitted by terminal 1 and 2, respectively. In the first time slot, each terminal precodes its symbols with matrix $P_{i}\in C^{N_{T,i}\times N_{S,i}}$, for i∈{1,2}, before transmitting it to the relays, which thus receive $y_{k}=\sum_{i=1}^{2}H_{i,k}P_{i}\;s_{i}+w_{k}$, for $k\in \{1,2,\ldots ,N_{C}\}$, where $H_{i,k}\in C^{N_{R}\times N_{T,i}}$ is the *first-hop* channel matrix (from terminal *i* to relay *k*), and $w_{k}\in C^{N_{R}}$ models additive noise at *k*th relay. By defining $y≜{\left[y_{1}^{T},y_{2}^{T},\ldots ,y_{N_{C}}^{T}\right]}^{T}\in C^{N_{C}N_{R}}$, the overall signal received by the relays can be compactly written as

(1) $$y=\sum_{i=1}^{2}H_{i}P_{i}\;s_{i}+w,$$

where $H_{i}≜{\left[H_{i,1}^{T},\;H_{i,2}^{T}\ldots ,\;H_{i,N_{C}}^{T}\right]}^{T}\in C^{N_{C}N_{R}\times N_{T,i}}$ gathers all first-hop channels and the vector $w≜{\left[w_{1}^{T},w_{2}^{T},\ldots ,w_{N_{C}}^{T}\right]}^{T}\in C^{N_{C}N_{R}}$ gathers all the noise samples.

In the second time slot, the *k*th relay forwards its received signal $y_{k}\in C^{N_{R}}$, by using the relaying matrix $F_{k}\in C^{N_{R}\times N_{R}}$, thus transmitting $z_{k}=F_{k}\;y_{k}$. The received signal at each terminal can be written, for i∈{1,2}, as 

(2) $$r_{i}=\sum_{k=1}^{N_{C}}G_{i,k}F_{k}\;y_{k}+n_{i}=G_{i}\;F\;y+n_{i},$$

where $G_{i,k}\in C^{N_{T,i}\times N_{R}}$ is the *second-hop* channel matrix (from relay *k* to terminal *i*), and the vector $n_{i}\in C^{N_{T,i}}$ is additive noise at terminal *i*. Additionally, we have defined in (2) the extended matrices $G_{i}≜[G_{i,1},\;G_{i,2}\ldots ,\;G_{i,N_{C}}]\in C^{N_{T,i}\times N_{C}N_{R}}$ and $F≜\mathrm{diag}\left(F_{1},F_{2},\ldots ,F_{N_{C}}\right)\in C^{N_{C}N_{R}\times N_{C}N_{R}}$. Moreover, by taking into account (1), the vector $r_{i}$ can also be directly written in terms of $s_{1}$ and $s_{2}$ as

(3) $$r_{i}=\sum_{j=1}^{2}C_{i,j}\;s_{j}+v_{i},$$

where $C_{i,j}≜G_{i}\;F\;H_{j}\;P_{j}\in C^{N_{T,i}\times N_{S,j}}$ is the *dual-hop* matrix from terminal *j* to *i*, for i,j∈{1,2}, and  vector $v_{i}≜G_{i}\;F\;w+n_{i}\in C^{N_{T,i}}$ is the overall noise.

We assume customarily [14,18] that each terminal can estimate and subtract the self-interference deriving from its own symbols. To do this, terminal *i* has to first acquire the matrix $C_{i,i}$, which can be obtained by resorting to standard training-based identification methods. Specifically, each data transmission can be preceded by a training period, wherein the two terminals transmit orthogonal pilot sequences to the relays. In this case, by redefining $r_{i}$ with a slight abuse of notation as $r_{i}-C_{i,i}\;s_{i}$, for i∈{1,2}, we write explicitly

(4) $$r_{i}=C_{i,\underline{i}}\;s_{\underline{i}}+v_{i}=G_{i}FH_{\underline{i}}\;P_{\underline{i}}\;s_{\underline{i}}+v_{i},$$

where $\underline{i}=2$ when i=1, whereas $\underline{i}=1$ when i=2.

At terminal *i*, vector $r_{i}$ is subject to linear equalization through matrix $D_{i}\in C^{N_{S,\underline{i}}\times N_{T,i}}$, thus yielding a soft estimate ${\hat{s}}_{\underline{i}}≜D_{i}\;r_{i}$ of the symbols $s_{\underline{i}}$ transmitted by terminal $\underline{i}$, whose entries are then subject to minimum-distance hard decision.

In the sequel, we consider the common assumptions: (a1) $s_{1}$ and $s_{2}$ are mutually independent zero-mean circularly symmetric complex (ZMCSC) random vectors, with $E\left[s_{i}\;s_{i}^{H}\right]=I_{N_{S,i}}$, for i∈{1,2}; (a2) the entries of $H_{i}$ and $G_{i}$ are independent identically distributed ZMCSC Gaussian unit-variance random variables, for i∈{1,2}; (a3) the noise vectors w, $n_{1}$ and $n_{2}$ are mutually independent ZMCSC Gaussian random vectors, statistically independent of ${\left\{s_{i},\;H_{i},\;G_{i}\right\}}_{i=1}^{2}$, with $E\left[ww^{H}\right]=\sigma_{w}^{2}\;I_{N_{C}N_{R}}$ and $E\left[n_{i}n_{i}^{H}\right]=\sigma_{n,i}^{2}\;I_{N_{T,i}}$, for i∈{1,2}.

Full channel-state information (CSI) is assumed to be available at both the terminals and the relays. Particularly, we assume that: (i) ${\left\{H_{i}\right\}}_{i=1}^{2}$ are known at the terminals and at the relays; (ii) the *k*th second-hop channel matrices $G_{1,k}$ and $G_{2,k}$ are known only to the *k*th relay, for $k\in \{1,2,\ldots ,N_{C}\}$; (iii) the dual-hop channel matrix $\left\{C_{i,\underline{i}}\right\}$ and the covariance matrix

(5) $$K_{v_{i}v_{i}}≜E\left[v_{i}\;v_{i}^{H}\right]=\sigma_{w}^{2}\;G_{i}\;F\;F^{H}\;G_{i}^{H}+\sigma_{n,i}^{2}\;I_{N_{T,i}}$$

of $v_{i}$ are known at the *i*th terminal, for i∈{1,2}. It should be noted that, hereinafter, all the ensemble averages are evaluated for fixed values of the first- and second-hop channel matrices.

## 3. The Proposed Closed-Form Design

With reference to model (4), the problem at hand is to find optimal values of ${\left\{P_{i}\right\}}_{i=1}^{2}$, F, and ${\left\{D_{i}\right\}}_{i=1}^{2}$ for recovering $s_{1}$ and $s_{2}$ according to a certain cost function and subject to suitable power constraints at the terminals and relays.

A common performance measure of the accuracy in recovering the symbol vector $s_{i}$ at terminal $\underline{i}$ is the mean-square value of the error $e_{i}≜{\hat{s}}_{i}-s_{i}$: ${\mathrm{MSE}}_{i}≜E[∥e_{i}{∥}^{2}]=\mathrm{tr}\left(K_{e_{i}e_{i}}\right)$, where $K_{e_{i}e_{i}}≜E\left[e_{i}e_{i}^{H}\right]$ is the error covariance matrix, which depends on $\left(P_{i},F,D_{\underline{i}}\right)$. As a global cost function for the overall two-way transmission, we consider as in References [15,16,17,18] the *sum-MSE*, defined as $\mathrm{MSE}\left({\left\{P_{i}\right\}}_{i=1}^{2},F,{\left\{D_{i}\right\}}_{i=1}^{2}\right)={\mathrm{MSE}}_{1}+{\mathrm{MSE}}_{2}$. It is well-known that, for fixed values of ${\left\{P_{i}\right\}}_{i=1}^{2}$ and F, the matrices ${\left\{D_{i}\right\}}_{i=1}^{2}$ minimizing the sum-MSE are the Wiener filters

(6) $$D_{i,\mathrm{mmse}}=C_{i,\underline{i}}^{H}{\left(C_{i,\underline{i}}\;C_{i,\underline{i}}^{H}+K_{v_{i}v_{i}}\right)}^{-1}$$

for i∈{1,2}, thus yielding

(7) $$\begin{array}{cc} \mathrm{MSE}({\left\{P_{i}\right\}}_{i=1}^{2},F) & ≜\mathrm{MSE}({\left\{P_{i}\right\}}_{i=1}^{2},F,{\left\{D_{i,\mathrm{mmse}}\right\}}_{i=1}^{2})\\ & =\sum_{i=1}^{2}\mathrm{tr}[(I_{N_{S,i}}+C_{\underline{i},i}^{H}K_{v_{\underline{i}}v_{\underline{i}}}^{-1}C_{\underline{i},i}{)}^{-1}]\;. \end{array}$$

It is noteworthy that the variables $P_{1}$, $P_{2}$, and F are coupled in (7) and, hence, the two terms in (7) cannot be minimized independently. Herein, we relax the original problem so as to *separate* the minimization of the two terms in (7).

As a first step, we observe that minimizing (7) is complicated by the presence of $K_{v_{\underline{i}}v_{\underline{i}}}^{-1}$, which depends non-trivially on F. For such a reason, we consider instead minimization of the following high signal-to-noise ratio (SNR) approximation:

(8) $$\mathrm{MSE}\left({\left\{P_{i}\right\}}_{i=1}^{2},F\right)\approx \sum_{i=1}^{2}\mathrm{tr}\left[{\left(I_{N_{S,i}}+\sigma_{n,\underline{i}}^{-2}\;C_{\underline{i},i}^{H}C_{\underline{i},i}\right)}^{-1}\right],$$

which turns out to be accurate when $\sigma_{w}^{2}\ll \min\left(\sigma_{n,\underline{i}}^{2},\mu_{\min}\right)$, where $\mu_{\min}$ is the smallest eigenvalue of $G_{\underline{i}}\;F\;F^{H}\;G_{\underline{i}}^{H}$. Suitable constraints must be set to avoid trivial solutions in minimizing (8). It is customary to impose power constraints to limit the average transmit power at the terminals:

(9) $$E[∥P_{i}s_{i}{∥}^{2}]=\mathrm{tr}\left(P_{i}\;P_{i}^{H}\right)\leq P_{T,i}>0$$

for i∈{1,2}. In order to limit F, we impose a constraint on the average power received at the terminals in the second time slot, that is, with reference to (2), we attempt to limit, for i∈{1,2}, the following quantities:

(10) $$E[∥G_{i}F\;y{∥}^{2}]=\mathrm{tr}\left(G_{i}FK_{yy}F^{H}G_{i}^{H}\right),$$

where $K_{yy}≜E\left[yy^{H}\right]=\sum_{i=1}^{2}H_{i}P_{i}P_{i}^{H}H_{i}^{H}+\sigma_{w}^{2}I_{N_{C}N_{R}}$ is the covariance matrix of y. It is noteworthy that (10) is typically limited in those scenarios where a target performance has to be achieved and per-node fairness is not of concern [10,12]. Moreover, the average power received at the terminals is an important metric measuring the human exposure to radio frequency (RF) fields generated by transmitters operating at mmWave frequencies [21] and, with respect to traditional per-relay transmit power constraints, it is more easily related to regulatory specifications [22]. To simplify (10), we exploit the following chain of inequalities:

(11) $$\begin{array}{cc} & \mathrm{tr}\left(G_{i}FK_{yy}F^{H}G_{i}^{H}\right)\leq \mathrm{tr}\left(G_{i}FF^{H}G_{i}^{H}\right)\mathrm{tr}\left(K_{yy}\right)\\ & \leq \mathrm{tr}\left(G_{i}FF^{H}G_{i}^{H}\right)\left[\sum_{i=1}^{2}\mathrm{tr}\left(H_{i}H_{i}^{H}\right)P_{T,i}+\sigma_{w}^{2}N_{C}N_{R}\right]\\ & ≲\mathrm{tr}\left(G_{i}FF^{H}G_{i}^{H}\right)N_{C}N_{R}\left(\sum_{i=1}^{2}N_{T,i}\;P_{T,i}+\sigma_{w}^{2}\right), \end{array}$$

where the last approximate inequality holds noting that, for fixed values of $N_{T,i}$, by the law of large numbers one has $H_{i}^{H}\;H_{i}/\left(N_{C}N_{R}\right)\to I_{N_{T,i}}$ almost surely as $N_{C}N_{R}$ gets large. Therefore, if we impose $\mathrm{tr}\left(G_{i}FF_{H}G_{i}^{H}\right)\leq {\tilde{P}}_{R,i}>0$, we get the upper bound:

(12) $$\mathrm{tr}\left(G_{i}FK_{yy}F^{H}G_{i}^{H}\right)≲\underset{≜P_{R,i}}{\underbrace{{\tilde{P}}_{R,i}N_{C}N_{R}\left(\sum_{i=1}^{2}N_{T,i}P_{T,i}+\sigma_{w}^{2}\right)}}\;.$$

Such a choice allows one to considerably simplify the system design. In summary, the optimization problem to be solved can be expressed as

(13) $$\begin{array}{cc} \min_{{\left\{P_{i}\right\}}_{i=1}^{2},F} & \sum_{i=1}^{2}\mathrm{tr}\left[{\left(I_{N_{S,i}}+\sigma_{n,\underline{i}}^{-2}\;C_{\underline{i},i}^{H}C_{\underline{i},i}\right)}^{-1}\right]\\ & s.\mathrm{to}\;\left\{\begin{array}{c} \mathrm{tr}\left(P_{i}\;P_{i}^{H}\right)\leq P_{T,i}\\ \mathrm{tr}\left(G_{\underline{i}}FF^{H}G_{\underline{i}}^{H}\right)\leq {\tilde{P}}_{R,\underline{i}} \end{array}\right.i\in \left\{1,2\right\}\;. \end{array}$$

In order to find a closed-form solution of (13), we introduce the matrix $B_{i}≜G_{i}F\in C^{N_{T,i}\times N_{C}N_{R}}$, with i∈{1,2}, and rewrite (13) as follows

(14) $$\begin{array}{cc} \min_{{\left\{P_{i}\right\}}_{i=1}^{2},{\left\{B_{i}\right\}}_{i=1}^{2}} & \sum_{i=1}^{2}\mathrm{tr}\left[{\left(I_{N_{S,i}}+\sigma_{n,\underline{i}}^{-2}\;P_{i}^{H}H_{i}^{H}B_{\underline{i}}^{H}B_{\underline{i}}H_{i}P_{i}\right)}^{-1}\right]\\ & s.\mathrm{to}\;\left\{\begin{array}{c} \mathrm{tr}\left(P_{i}\;P_{i}^{H}\right)\leq P_{T,i}\\ \mathrm{tr}\left(B_{\underline{i}}B_{\underline{i}}^{H}\right)\leq {\tilde{P}}_{R,\underline{i}} \end{array}\right.i\in \left\{1,2\right\}\;. \end{array}$$

Remarkably, the cost function is the sum of two terms: the former one depends only on the variables $\left\{P_{1},B_{2}\right\}$, whereas the latter one involves only the variables $\left\{P_{2},B_{1}\right\}$. Therefore, (14) can be decomposed in two problems involving $\left\{P_{1},B_{2}\right\}$ and $\left\{P_{2},B_{1}\right\}$ separately, which can be solved in parallel in a closed-form manner. Indeed, capitalizing on such a decomposition, the solution of (14) can be characterized by the following theorem.

**Theorem** **1.** *Assume that: (a4) $P_{i}\in C^{N_{T,i}\times N_{S,i}}$ is full-column rank, that is, $\mathrm{rank}\left(P_{i}\right)=N_{S,i}\leq N_{T,i}$, i∈{1,2}; (a5) $B_{\underline{i}}H_{i}\in C^{N_{T,\underline{i}}\times N_{T,i}}$ is full-column rank, that is, $\mathrm{rank}\left(B_{\underline{i}}H_{i}\right)=N_{T,i}\leq N_{T,\underline{i}}$, for i∈{1,2}. Moreover, let $H_{i}=H_{h,i}\Lambda_{h,i}V_{h,i}^{H}$ denote the singular value decomposition (SVD) of $H_{i}$, where $U_{h,i}\in C^{N_{C}N_{R}\times N_{C}N_{R}}$ and $V_{h,i}\in C^{N_{T,i}\times N_{T,i}}$ are the unitary matrices of left/right singular vectors, and $\Lambda_{h,i}\in C^{N_{C}N_{R}\times N_{T,i}}$ is the rectangular diagonal matrix of the corresponding singular values arranged in increasing order. Then, the solution of (*14*) has the following general form:*

(15) $$\begin{array}{cc} P_{i} & =V_{h,i,\mathrm{right}}\;\Omega_{i} \end{array}$$

(16) $$\begin{array}{cc} B_{\underline{i}} & =Q_{\underline{i}}\;\Delta_{\underline{i}}U_{h,i,\mathrm{right}}^{H}, \end{array}$$

*where $V_{h,i,\mathrm{right}}$ contains the $N_{S,i}$ rightmost columns of $V_{h,i}$, $U_{h,i,\mathrm{right}}$ contains the $N_{T,i}$ rightmost columns of $U_{h,i}$, the diagonal matrices $\Omega_{i}\in R^{N_{S,i}\times N_{S,i}}$ and $\Delta_{\underline{i}}\in R^{N_{T,i}\times N_{T,i}}$ will be specified soon after, and $Q_{\underline{i}}\in C^{N_{T,\underline{i}}\times N_{T,i}}$ is an arbitrary semi-unitary matrix, that is, $Q_{\underline{i}}^{H}Q_{\underline{i}}=I_{N_{T,i}}$.*

**Proof.**  See Appendix A. □

**Remark 1.** *(a4) implies that $N_{S,i}\leq N_{T,i}$, i∈{1,2}.*

**Remark 2.** *(a5) implies that $H_{i}$ is full-column rank too, that is, $\mathrm{rank}\left(H_{i}\right)=N_{T,i}$ and $N_{T,1}=N_{T,2}$. Hence, in the following we set $N_{T}≜N_{T,1}=N_{T,2}$.*

Under (a4) and (a5), the dual-hop channel matrices ${\left\{C_{i,\underline{i}}=B_{i}H_{\underline{i}}P_{\underline{i}}\right\}}_{i=1}^{2}$ are full-column rank, that is, $\mathrm{rank}\left(C_{i,\underline{i}}\right)=N_{S,\underline{i}}\leq N_{T,\underline{i}}$, for i=1,2: this ensures perfect recovery of the source symbol vectors ${\left\{s_{i}\right\}}_{i=1}^{2}$ at the terminals in the absence of noise by means of linear equalizers. Although Theorem 1 holds for any value of $N_{S,1}$ and $N_{S,2}$, we will assume herein that $N_{S,1}=N_{S,2}=N_{T}$, which allows the terminals to transmit as many symbols as possible with an acceptable performance in practice.

Theorem 1 allows one to rewrite the optimization problem (14) in a simpler scalar form:

(17) $$\begin{array}{cc} & \min_{\begin{array}{c} {\left\{z_{1,\ell },w_{2,\ell }\right\}}_{\ell =1}^{N_{T}}\\ {\left\{z_{2,\ell },w_{1,\ell }\right\}}_{\ell =1}^{N_{T}} \end{array}}\sum_{i=1}^{2}\sum_{\ell =1}^{N_{T}}\frac{1}{1+\sigma_{n,\underline{i}}^{-2}\;\lambda_{\ell }^{2}\left(H_{i}\right)z_{i,\ell }\;w_{\underline{i},\ell }}\\ & s.\mathrm{to}\;\left\{\begin{array}{cc} \sum_{\ell =1}^{N_{T}}z_{i,\ell }\leq P_{T,i}\\ \sum_{\ell =1}^{N_{T}}w_{\underline{i},\ell }\leq {\tilde{P}}_{R,\underline{i}}\\ w_{\underline{i},\ell },z_{i,\ell }>0 & \forall \ell \in \{1,2,\ldots ,N_{S,i}\} \end{array}\right.i\in \left\{1,2\right\}, \end{array}$$

with $z_{i,\ell }$ and $w_{\underline{i},\ell }$ representing the *ℓ*th squared diagonal entry of $\Omega_{i}$ and $\Delta_{\underline{i}}$, respectively, whereas $\lambda_{\ell }\left(H_{i}\right)$ denotes the *ℓ*th nonzero singular value of $H_{i}$, for $\ell \in \{1,2,\ldots ,N_{T}\}$. Similarly to (14), problem (17) can be decomposed into two separate problems involving disjoint subsets of variables.

It can be shown, with straightforward manipulations, that the objective function in (17) is convex if and only if

(18) $$z_{i,\ell }\;w_{\underline{i},\ell }\geq \frac{\sigma_{n,\underline{i}}^{2}}{3\lambda_{\ell }^{2}(H_{i})}$$

$\forall \ell \in \{1,2,\ldots ,N_{S,i}\}$, with i∈{1,2}. It is also seen that, based on (a2), one has $\lambda_{\min}\left(H_{i}\right)\gg 1$ in the large $N_{C}N_{R}$ limit, with i∈{1,2}. Thus, condition (18) boils down to $z_{i,\ell },\;w_{\underline{i},\ell }>0$, for all $\ell \in \{1,2,\ldots ,N_{S,i}\}$, with i∈{1,2}, which is already included in the constraints of (17). Therefore, convex programming can be used to find a global minimum of (17).

To calculate the relaying matrices, let us partition solution (16) as $B_{\underline{i}}=\left[B_{\underline{i},1},B_{\underline{i},2},\cdots ,B_{\underline{i},N_{C}}\right]$, with $B_{\underline{i},k}\in C^{N_{T}\times N_{R}}$, i∈{1,2}. Defining ${\tilde{G}}_{k}≜[G_{1,k}^{T},\;G_{2,k}^{T}{]}^{T}\in C^{2N_{T}\times N_{R}}$ and ${\tilde{B}}_{k}≜{\left[B_{1,k}^{T},B_{2,k}^{T}\right]}^{T}\in C^{2N_{T}\times N_{R}}$, and assuming that ${\tilde{G}}_{k}$ is full-row rank, that is, $\mathrm{rank}\left({\tilde{G}}_{k}\right)=2N_{T}\leq N_{R}$, with $k\in \{1,2,\ldots ,N_{C}\}$, the *k*th relay can construct its own relaying matrix by solving the matrix equation ${\tilde{G}}_{k}F_{k}={\tilde{B}}_{k}$, whose minimum-norm solution is given by

(19) $$F_{k}={\tilde{G}}_{k}^{†}{\tilde{B}}_{k},$$

where the superscript † denotes the Moore-Penrose inverse.    
**Algorithm 1:** The proposed design algorithm. Input quantities: $\{H_{i},\;G_{i},\;\sigma_{n,i}^{2},P_{T,i},\;{\tilde{P}}_{R,i}{\}}_{i=1}^{2}$ Output quantities: ${\left\{P_{i},D_{i,\mathrm{mmse}}\;\right\}}_{i=1}^{2}$ and ${\left\{F_{k}\right\}}_{k=1}^{N_{C}}$
  1.Choose arbitrary ${\left\{Q_{i}\right\}}_{i=1}^{2}$ such that $Q_{i}^{H}Q_{i}=I_{N_{S,\underline{i}}}$.  2.Perform the SVD of $\{H_{i}{\}}_{i=1}^{2}$. and collect the ${\left\{N_{S,i}\right\}}_{i=1}^{2}$ largest singular values and the corresponding left/right singular vectors.  3.Solve the convex problem (17) in the disjoint subsets of variables ${\left\{z_{1,\ell },\;w_{2,\ell }\right\}}_{\ell =1}^{N_{S,1}}$ and ${\left\{z_{2,\ell },\;w_{1,\ell }\right\}}_{\ell =1}^{N_{S,2}}$ separately.  4.From the solution of step 3, build the matrices ${\left\{\Omega_{i},\Delta_{i}\right\}}_{i=1}^{2}$.  5.Build the matrices ${\left\{P_{i},\;B_{i}\right\}}_{i=1}^{2}$ according to (15) and (16).  6.Calculate ${\left\{F_{k}\right\}}_{k=1}^{N_{C}}$ according to (19).  7.Calculate ${\left\{D_{i,\mathrm{mmse}}\right\}}_{i=1}^{2}$ according to (6).

With reference to the step-by-step description of the proposed design algorithm reported at the top of this page, the following comments are in order. The convex optimization in step 3 can be efficiently carried out using standard techniques, such as the interior-point method. We observe that the worst-case theoretical complexity of the interior-point method is proportional to $\sqrt{N_{T}}$. Hence, for a realistic setting of the system parameters, the computational complexity of the proposed algorithm, is dominated by the SVD computation (in step 2), which is of order $O(N_{C}\;N_{R}\;N_{T}^{2})$ and, thus, it *linearly* grows with the number $N_{C}$ of relays. It is noteworthy that, even though the alternating algorithm proposed in Reference [16] allows to solve a nonconvex problem by solving convex subproblems, it is more complex than calculating the solution of (17); moreover, it requires proper initialization to monotonically converge to (at least) a local optimum.

## 4. Simulation Results

In this section, to assess the performance of the considered design, we present the results of Monte Carlo computer simulations, aimed at evaluating the average (with respect to channel realizations) bit-error-rate (BER) of the proposed cooperative two-way MIMO system. We consider a network encompassing two terminals equipped with $N_{T}=2$ antennas, and transmitting QPSK symbols with $N_{S,1}=N_{S,2}=2$. The $N_{C}$ relays are equipped with $N_{R}=4$ antennas. We also assume that $P_{T,1}=P_{T,2}=P_{k}=P$, for all $k\in \{1,2,\ldots ,N_{C}\}$, where $P_{k}$ represents the average transmitted power at the *k*th relay, and set $\sigma_{w}^{2}=\sigma_{n,1}^{2}=\sigma_{n,2}^{2}=1$. Consequently, the energy per bit to noise power spectral density ratio $E_{b}/N_{0}$ is a measure of the per-antenna link quality of both the first- and second-hop transmissions. The BER is evaluated by carrying out $10^{3}$ independent Monte Carlo trials, with each run using independent sets of channel realizations and noise, and an independent record of $10^{6}$ source symbols.

We compare the performances of our design (labeled as “Proposed”) to those of the iterative technique proposed in Reference [16], which has been shown [16] in its turn to outperform other iterative techniques, such as the gradient-descent technique of Reference [15]. It is worthwhile to note that both the strategies under comparison require the same amount of CSI. Furthermore, since the method of Reference [16] imposes different power constraints on the design of the relaying matrices, our solutions for ${\left\{F_{k}\right\}}_{k=1}^{N_{C}}$ are properly scaled so as to ensure that the average power transmitted by each relay is the same for both methods.

**Figure 2:**
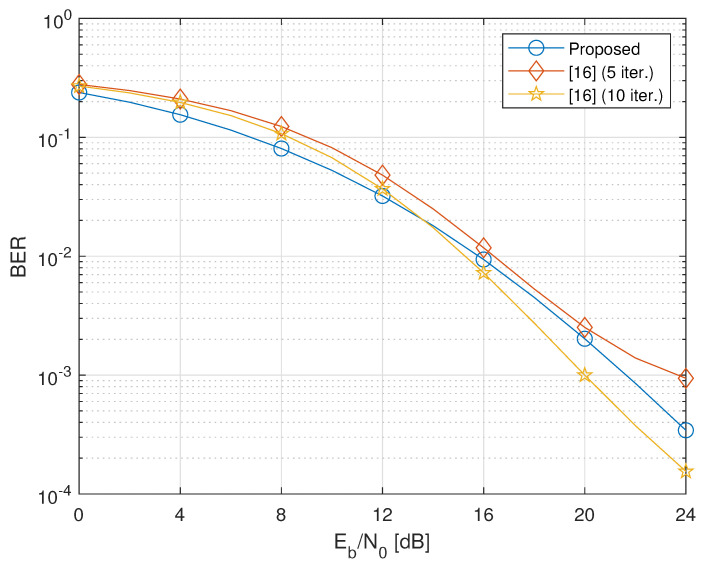
Bit-error-rate (BER) versus $E_{b}/N_{0}$ of the proposed design versus the iterative method of Reference [16] ($N_{C}=2$).

**Figure 3:**
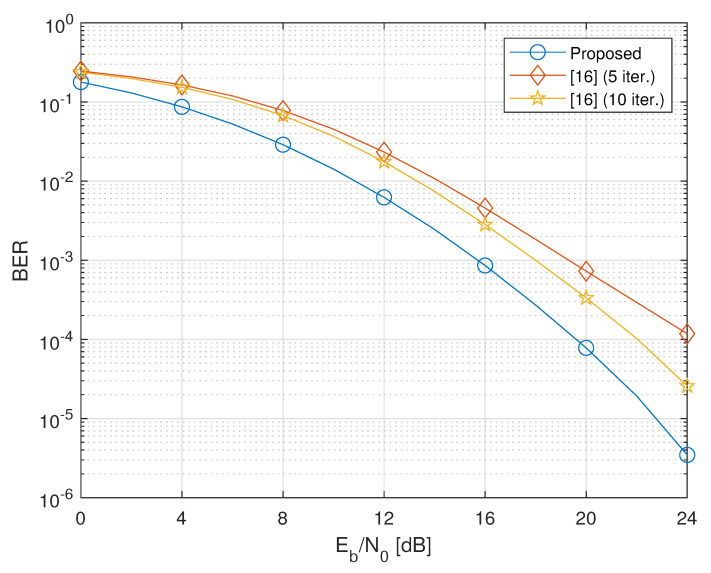
BER versus $E_{b}/N_{0}$ of the proposed design versus the iterative method of Reference [16] ($N_{C}=3$).

**Figure 4:**
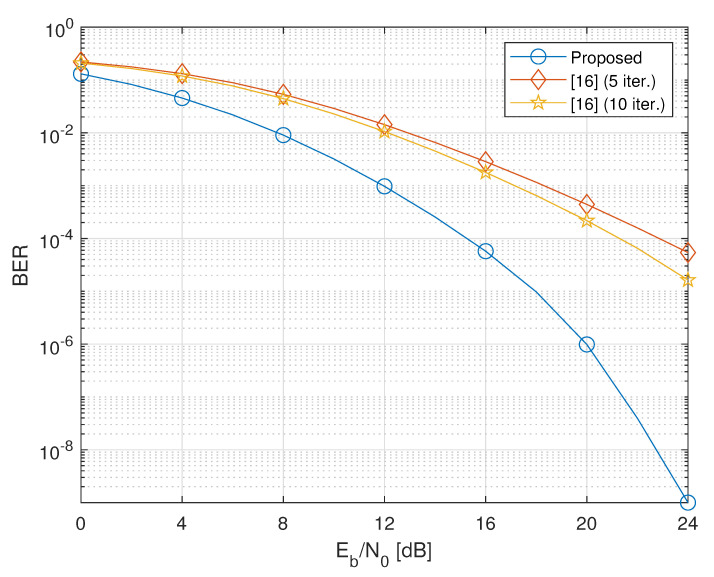
BER versus $E_{b}/N_{0}$ of the proposed design versus the iterative method of Reference [16] ($N_{C}=4$).

In Figure 2, Figure 3 and Figure 4, we report the BER for different values of the number $N_{C}\in \left\{2,3,4\right\}$ of relays. Results in Figure 2 for $N_{C}=2$ show that the proposed closed-form design, based on the solution of the relaxed problem (14), exhibits performances comparable with the iterative solution of Reference [16] in the considered range of $E_{b}/N_{0}$ values only when the latter employs more than 5 iterations. Specifically, when the method of Reference [16] employs 10 iterations, a crossover can be observed in Figure 2 between the BER curve of the proposed algorithm and that of Reference [16]. This behavior is due to the fact that the rate of convergence of Reference [16] strongly depends on the SNR.

Figure 3 and Figure 4 show that, as the number of relays increases, the proposed method clearly outperforms the method of Reference [16] even when the latter employs 10 iterations. Performance improvement of Reference [16] is negligible after 10 iterations.

In a nutshell, although the alternating iterative procedure [16] attempts to solve the nonconvex original two-way constrained minimum sum-MSE problem, its convergence behaviors are affected in practice by both the operative SNR and number of relays: in the low-SNR region and/or when the number of relays is sufficiently large, convergence to a local minimizer is not guaranteed in a reasonable number of iterations for all possible initializations. This is the price to pay for swapping a difficult joint optimization with a sequence of easier problems involving subsets of the variables. On the other hand, the proposed optimization strategy gives up the idea of solving the original nonconvex problem, by resorting to suitable relaxations of both the cost function and the relaying power constraint. This allows us to jointly optimize all the variables, without using burdensome iterative algorithms.

## 5. Discussion and Directions for Future Work

We tackled the joint sum-MSE design of terminal precoders/decoders and relay forwarding matrices for two-way AF MIMO 5G systems. We showed that a relaxed version of such a problem can be separated into two simpler ones, which can be solved in parallel by admitting closed-form solutions. The proposed technique exhibits a performance gain over the iterative method of Reference [16], exhibiting a better scaling with the number of relays and a reduced computational complexity.

In this paper, we assumed the availability of full-CSI at both terminals and the relays. In this respect, an interesting research subject consists of considering the use of partial CSI to extend network lifetime and reduce the complexity burden. Moreover, since channel estimation errors occur in practical situations, an additional research issue is to develop robust optimization designs. 

## Appendix A. Proof of Theorem 1

We focus on the optimization (14) with indexes i=1 and $\underline{i}=2$, that is, we consider

(A1) $$\begin{array}{cc} \min_{P_{1},B_{2}}\; & \mathrm{tr}[{\left(I_{N_{S,1}}+\sigma_{n,2}^{-2}\;P_{1}^{H}H_{1}^{H}B_{2}^{H}B_{2}H_{1}P_{1}\right)}^{-1}]\\ & s.\mathrm{to}\;\left\{\begin{array}{c} \mathrm{tr}\left(P_{1}\;P_{1}^{H}\right)\leq P_{T,1}\\ \mathrm{tr}\left(B_{2}B_{2}^{H}\right)\leq {\tilde{P}}_{R,2} \end{array}\right.. \end{array}$$

We note that under (a4) and (a5), one has $\mathrm{rank}\left(B_{2}H_{1}P_{1}\right)=N_{S,1}\leq N_{T,1}$. Let $U_{a}\Lambda_{a}U_{a}^{H}$ be the eigenvalue decomposition (EVD) of $A≜H_{1}^{H}B_{2}^{H}B_{2}H_{1}\in C^{N_{T,1}\times N_{T,1}}$, where the diagonal matrix $\Lambda_{a}\in R^{N_{T,1}\times N_{T,1}}$ and the unitary matrix $U_{a}\in C^{N_{T,1}\times N_{T,1}}$ collect the eigenvalues, arranged in increasing order, and the eigenvectors of A, respectively. The objective function in (A1) is a Schur-concave function of the diagonal elements of ${\left(I_{N_{S,1}}+\sigma_{n,2}^{-2}\;P_{1}^{H}AP_{1}\right)}^{-1}$. In this case, it can be shown [23] that there is an optimal $P_{1}$ such that $P_{1}^{H}AP_{1}$ is diagonal, whose diagonal elements are assumed to be arranged in increasing order, and such an optimal matrix, which also minimizes $\mathrm{tr}(P_{1}P_{1}^{H})$, is given by

(A2) $$P_{1}=U_{a,\mathrm{right}}\Omega_{1},$$

where $U_{a,\mathrm{right}}\in C^{N_{T,1}\times N_{S,1}}$ contains the $N_{S,1}\leq N_{T,1}$ rightmost columns from $U_{a}$, and $\Omega_{1}\in C^{N_{S,1}\times N_{S,1}}$ is a diagonal matrix. Let $Q_{2}\in C^{N_{T,2}\times N_{T,1}}$ be an arbitrary semi-unitary matrix, that is, $Q_{2}^{H}Q_{2}=I_{N_{T,1}}$, it follows from the EVD of the matrix A that $B_{2}H_{1}=Q_{2}\Lambda_{a}^{1/2}U_{a}^{H}$. Noting that $\mathrm{rank}\left(H_{1}\right)=N_{T,1}$, by substituting the ordered SVD of $H_{1}=U_{h,1}\Lambda_{h,1}V_{h,1}^{H}$ in this equation, after some algebraic manipulations, one has that the minimum-norm solution [24] of the matrix equation $B_{2}U_{h,1}\Lambda_{h,1}=Q_{2}\Lambda_{a}^{1/2}U_{a}^{H}V_{h,1}$ is

(A3) $$B_{2}=Q_{2}\Lambda_{a}^{1/2}{\tilde{U}}_{a}\Lambda_{h,1,\mathrm{right}}^{-1}U_{h,1,\mathrm{right}},$$

where $U_{h,1,\mathrm{right}}$ collects the $N_{T,1}$ rightmost columns of $U_{h,1}$, whereas ${\tilde{U}}_{a}≜U_{a}^{H}V_{h,1}\in C^{N_{T,1}\times N_{T,1}}$ and the diagonal a $\Lambda_{h,1,\mathrm{right}}\in R^{N_{T,1}\times N_{T,1}}$ gathers the $N_{T,1}$ nonzero singular values of $H_{1}$ in increasing order. The aim is now to further determine (A3) by properly choosing ${\tilde{U}}_{a}$ such that $\mathrm{tr}\left(B_{2}B_{2}^{H}\right)=\mathrm{tr}\left[\left({\tilde{U}}_{a}\Lambda_{h,1,\mathrm{right}}^{-2}{\tilde{U}}_{a}\right)\Lambda_{a}\right]$ has the smallest value. It is readily seen that $\mathrm{tr}(B_{2}B_{2}^{H})$ is invariant to the choice of $Q_{2}$. By observing that ${\tilde{U}}_{a}^{H}{\tilde{U}}_{a}=I_{N_{T,1}}$ and using a known trace inequality, one has

(A4) $$\mathrm{tr}\left[\left({\tilde{U}}_{a}\Lambda_{h,1,\mathrm{right}}^{-2}{\tilde{U}}_{a}\right)\Lambda_{a}\right]\geq \sum_{\ell =1}^{N_{T,1}}\lambda_{h,1,\ell }^{-2}\lambda_{a,\ell },$$

where $\lambda_{h,1,\ell }$ and $\lambda_{a,\ell }$ denote the *ℓ*th diagonal entry of $\Lambda_{h,1,\mathrm{right}}$ and $\Lambda_{a}$, respectively. The equality in (A4) holds when

(A5) $${\tilde{U}}_{a}=U_{a}^{H}V_{h,1}=I_{N_{T,1}}.$$

Substituting (A5) in (A3), after some algebraic manipulations, one obtains $B_{2}=Q_{2}\;\Delta_{2}U_{h,1,\mathrm{right}}^{H}$, with $\Delta_{2}≜\Lambda_{a}^{1/2}\Lambda_{h,1,\mathrm{right}}^{-1}\in R^{N_{T,1}\times N_{T,1}}$. Solution (15) comes from substituting in (A2) the minimum-norm solution [24] of (A5), that is, $U_{a}=V_{h,1,\mathrm{right}}$.
